# Measuring the impact of an integrated bite case management program on the detection of canine rabies cases in Vietnam

**DOI:** 10.3389/fpubh.2023.1150228

**Published:** 2023-10-18

**Authors:** Yasmeen B. Ross, Chuong Dinh Vo, Sarah Bonaparte, Minh Quang Phan, Diep Thi Nguyen, Thin Xuan Nguyen, Thanh Tat Nguyen, Lillian Orciari, Tho Dang Nguyen, Oanh Kim Thi Nguyen, Trang Thuy Do, Anh Thi Phuong Dao, REACT Development Team, Andrew Luke Gamble, Fred Lohr, Luke Gamble, Ryan Wallace, Long Van Nguyen

**Affiliations:** ^1^National Center for Emerging and Zoonotic Infectious Diseases, Centers for Disease Control and Prevention, Atlanta, GA, United States; ^2^Department of Animal Health, Ministry of Agriculture and Rural Development, Hanoi, Vietnam; ^3^Phu Tho Provincial Sub Department of Animal Health, Phu Tho, Vietnam; ^4^Department of Animal Health, National Center for Veterinary Diagnostics, Hanoi, Vietnam; ^5^Division of Global Health Protection, Centers for Disease Control and Prevention, Hanoi, Vietnam

**Keywords:** rabies, surveillance system, One Health, Vietnam, dog, veterinary diagnostic

## Abstract

**Introduction:**

Dog-mediated rabies is enzootic in Vietnam, resulting in at least 70 reported human deaths and 500,000 human rabies exposures annually. In 2016, an integrated bite cases management (IBCM) based surveillance program was developed to improve knowledge of the dog-mediated rabies burden in Phu Tho Province of Vietnam.

**Methods:**

The Vietnam Animal Rabies Surveillance Program (VARSP) was established in four stages: (1) Laboratory development, (2) Training of community One Health workers, (3) Paper-based-reporting (VARSP 1.0), and (4) Electronic case reporting (VARSP 2.0). Investigation and diagnostic data collected from March 2016 to December 2019 were compared with historical records of animal rabies cases dating back to January 2012. A risk analysis was conducted to evaluate the probability of a rabies exposure resulting in death after a dog bite, based on data collected over the course of an IBCM investigation.

**Results:**

Prior to the implementation of VARSP, between 2012 and 2015, there was an average of one rabies investigation per year, resulting in two confirmed and two probable animal rabies cases. During the 46 months that VARSP was operational (2016 – 2019), 1048 animal investigations were conducted, which identified 79 (8%) laboratory-confirmed rabies cases and 233 (22%) clinically-confirmed(probable) cases. VARSP produced a 78-fold increase in annual animal rabies case detection (one cases detected per year pre-VARSP vs 78 cases per year under VARSP). The risk of succumbing to rabies for bite victims of apparently healthy dogs available for home quarantine, was three deaths for every 10,000 untreated exposures.

**Discussion:**

A pilot IBCM model used in Phu Tho Province showed promising results for improving rabies surveillance, with a 26-fold increase in annual case detection after implementation of a One Health model. The risk for a person bitten by an apparently healthy dog to develop rabies in the absence of rabies PEP was very low, which supports the WHO recommendations to delay PEP for this category of bite victims, when trained animal assessors are available and routinely communicate with the medical sector. Recent adoption of an electronic IBCM system is likely to expedite adoption of VARSP 2.0 to other Provinces and improve accuracy of field decisions and data collection.

## 1. Introduction

Rabies is a viral disease that manifests as a progressive, fatal encephalitis. Worldwide, there are an estimated 59,000 human rabies deaths each year, and over 95% are due to dog bites (1, 2). Rabies is entirely preventable if vaccine and immunoglobulin are administered properly and promptly after an exposure. The canine rabies virus variant is enzootic in Asia and approximately 60% of all human rabies deaths occur in this region (1). In Vietnam, rabies control through human and animal vaccination and dog population management in urban areas have been successful. In recent years, 80–100 human rabies deaths are reported annually, compared to more than 400 deaths just a decade ago (3).

In 1996, Vietnam established the National Rabies Control Program to increase support and resources to expand post-exposure prophylaxis (PEP) centers, introduce and revise key legislation and guidelines, and improve One Health collaborations (3). The implementation of this rabies control program resulted in the increase of vaccination centers nationwide, leading to increased PEP availability. Surveillance systems established through the National Rabies Control Program found that over 100 suspected rabid dogs were found in 20 districts across 10 Provinces, from 2008 to 2014 (3). Dog owners have been required to register and vaccinate their dogs since 2017, and data modeling conducted in 2015 estimated the national vaccination coverage in Vietnam to be approximately 43% (3).

In Viet Tri city, Phu Ninh, Lam Thao, and Thanh Ba districts, and Phu Tho town (all located within Phu Tho Province, Vietnam), 15 dog-mediated human rabies deaths were reported from 2010 to 2013 (4). Phu Tho Province has a population of 1.4 million people and is located 100 km northwest of Hanoi. After recognition of the high rate of human rabies deaths in Phu Tho Province by government officials, the provincial Department of Agriculture and Rural Development, in partnership with the World Organization for Animal Health (WOAH) and the Food and Agriculture Organization of the United Nations (FAO), enhanced canine rabies vaccination in the province, increasing coverage from 23% in 2011 to 74% by 2014 (4, 5). Subsequently, reported human rabies deaths in the whole Province of Phu Thu declined to just two human deaths in 2015. Given the success in controlling human rabies deaths, this Province was chosen to pilot an Integrated Bite Case Management (IBCM) program with two goals in mind (1) improve surveillance for animal rabies to inform control measures and (2) to determine if the rabies risk in dogs that have bitten humans is low enough to justify a risk-based approach to providing PEP to dog bite victims.

Vietnam has invested significant resources into controlling rabies to work towards the global goal of zero human deaths from dog-mediated rabies by 2030 (3, 6). Barriers to successful canine rabies control in Vietnam include the lack of accurate dog population estimations, low canine vaccination coverage, lack of rabies awareness among community members, and limited canine rabies surveillance (3). Canine rabies surveillance plays an integral role in rabies control for several key reasons. Surveillance provides epidemiologic data to inform cost-effective control policies and enables monitoring and evaluation of control strategies. Surveillance is also integral for the rapid detection of animal rabies outbreaks. IBCM is a type of rabies surveillance that utilizes a One Health approach to identify suspected rabid animals and human exposures. IBCM has shown to provide additional community benefits, such as removing rabid animals to limit the enzootic rabies transmission cycle (7). IBCM programs also rely on community one health workers (COHWs) who actively seek out bite victims and provide risk assessments and PEP counseling, which can directly reduce human rabies deaths (8). Finally, routine and reliable IBCM programs, where case investigation outcomes are relayed to the patient and medical provider, can reduce the use of unnecessary rabies vaccination when an animal tests negative for the disease (8, 9).

In 2016, an IBCM rabies surveillance program was piloted to improve knowledge of the dog-mediated rabies burden in the Phu Tho Province of Vietnam. This article details the process of developing the program, data of animal rabies cases and bites reported through this program, and a comparison of rabies prevalence and characteristics of data collection methods to the pre-surveillance period.

## 2. Methods

The Vietnam Animal Rabies Surveillance Program (VARSP) was developed in 2016 under the leadership of Vietnam’s Department of Animal Health (DAH) in collaboration with the United States Center for Disease Control and Prevention (US CDC) to strengthen the animal health services of Vietnam capacity to detect, diagnose, and control dog-mediated rabies. VARSP was established in four stages from 2016 to 2019. Historical records for confirmed cases of rabies in Phu Tho, preceding the VARSP program, were available between January 2012 and January 2016.

### 2.1. Stage one: laboratory development

Prior to 2015, rabies diagnosis was only available within the Vietnam Ministry of Health, which conducted the Direct Fluorescent Antibody (DFA) test and reverse-transcriptase PCR (RTPCR) (10). Diagnostics were primarily conducted on suspected human rabies cases, but animal cases were tested upon request from the DAH. In March 2015, US CDC assisted in the establishment of a national rabies diagnostic facility capable of performing rabies testing using DFA at the National Center for Veterinary Diagnosis (NCVD) in Hanoi. Ten staff were trained on brain tissue removal from deceased animals, sample preparation, reagent management and optimization, and antigen detection by DFA. With financial support from the Global Health Securities Agenda, the laboratory was equipped with one fluorescent microscope, an incubator, freezer, fume hood, and supplies required for processing and diagnosing samples (11). Laboratorians were routinely assessed for DFA proficiency and required to receive rabies pre-exposure vaccination before conducting rabies diagnostic activities. In 2018 a second laboratory training took place, establishing capacity to perform real time reverse transcriptase PCR.

### 2.2. Stage two: training of community One Health workers

In August 2015, a two-day training was held for selected COHWs on the principles of rabies surveillance protocols, animal behavior and identifying rabid animals, animal capture and handling, and humane euthanasia for suspect rabid animals. This training included both classroom and live-animal fieldwork components. The eleven participants were current employees of Phu Tho Provincial Sub Department of Animal Health, Health Centers of Phu Ninh and Viet Tri, Veterinary stations of Viet Tri, Phu Ninh and Thanh Ba districts. Eligibility was based on knowledge of the animal health system in Vietnam, previous knowledge of infectious diseases, and ability to clearly communicate information about rabies to bite victims. All COHWs were required to be vaccinated against rabies.

### 2.3. Stage three: VARSP implementation

Vietnam is comprised of 63 Provinces and centrally-run municipalities which are then divided into districts (12). To establish a framework for rabies detection, control, and elimination, a community-based animal rabies surveillance program was created with a focus on five areas in Phu Tho Province: Phu Ninh (district), Thanh Ba (district), Lam Thao (district), Doan Hung (district) and Viet Tri (capital city Province). The total human population in this area in 2016, based on Phu Tho Province statistics records, is 621,356, with an estimated 109,935 dogs. The above mentioned areas make up a surveillance area that is geographically isolated by a river to the East, a river to the West, and mountains to the North. These geographical barriers make this region of the Phu Tho Province an ideal location to establish a rabies elimination program, as rabies transmission has been shown to be halted or delayed by geographical boundaries such as rivers, mountains, and sparsely populated areas (13). Furthermore, a dog vaccination program supported by WOAH and FAO in this Province was suspected to have significantly reduced dog-mediated rabies virus transmission; however limited surveillance had been conducted to confirm this suspicion (5).

VARSP case investigations were included in this analysis if they occurred between March 2016 and December 2019. Investigations were initiated when COHWs were alerted of a dog bite, suspected rabid animal, or a suspected human rabies exposure from the medical sector or other community cooperators. COHWs met with local community leaders to encourage community-based reporting and established formal information exchange processes with PEP clinics to obtain timely reports of suspected human rabies exposures. Formal information exchange occurred through a network of focal points communicating through telephone call or the social messaging platform Zalo. COHWs or the Provincial Rabies Epidemiologist (PRE) collected information about the location of the animals involved in the bite event and/or animals with symptoms or rabies. A unique patient identification number that links the health record to a bite investigation form was assigned.

**Figure 1:**
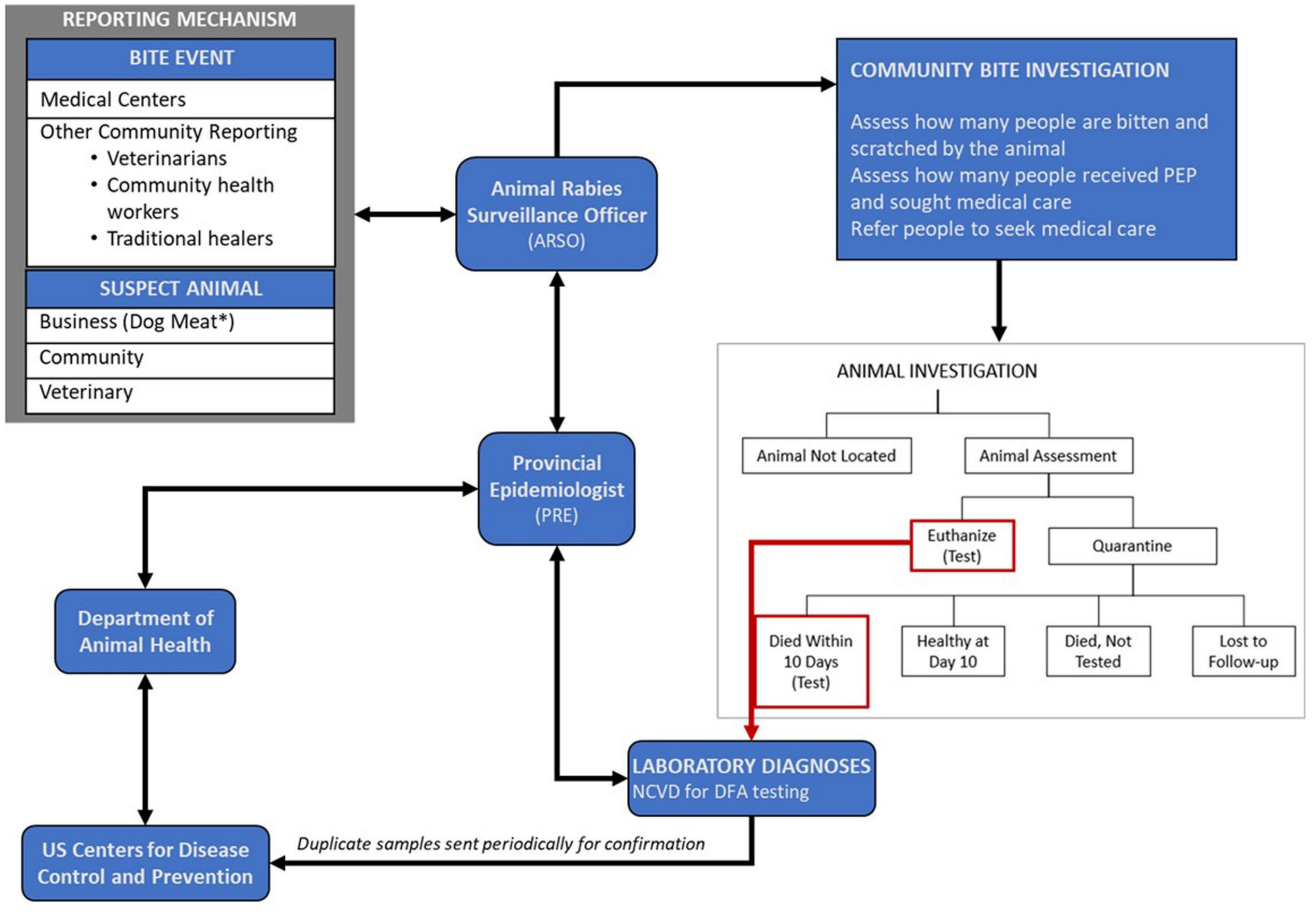
Vietnam Animal Rabies Surveillance Program structure for reporting, investigations, diagnostic testing of suspected rabid animals. Arrows represent the flow of information.

VARSP investigations were composed of two parts, community bite investigation and animal investigation, which were typically initiated within 24 h of notification (Figure 1). Animals that had an identifiable owner and appeared healthy were placed on a 10-day in-home quarantine. Animals healthy after 10 days were released from quarantine, and animals that displayed signs consistent with rabies during the investigation or the observation period were euthanized and tested at NCVD. American Veterinary Medical Association standards were used to adopt a local euthanasia protocol (Appendix). Investigation results were reported back to the dog owner, bite victim (s), medical center(s), and other relevant stakeholders so human medical treatment could be adjusted as necessary and appropriate epidemiologic interventions could be implemented. Additional persons identified to have been exposed during the course of the community bite investigations were referred to nearby medical facilities for PEP evaluation.

Data from investigations were documented on paper investigation forms which collected 55 variables including information on the overall health status of the animals, presenting clinical signs, health history, and human exposures (Supplementary material). This information was used for manual determination of the interim and final case status of the animal (non-case, suspect, probable, confirmed), PEP recommendations, and programmatic evaluation. From March 2016 until August 2019 VARSP operated solely as a paper-based collection system that was hand entered into an access database on a monthly basis and shared with Regional, National, and CDC on a quarterly basis.

### 2.4. Stage four: electronic data capture

In September 2019, IBCM focal points of the Phu Tho Provincial Sub Department of Animal Health (SDAH), district veterinary stations, and staff of Phu Tho provincial Center for Diseases Control (provincial CDC) attended a two-day training on the use of the Rabies Exposure Assessment and Contact Tracing (REACT) App (14). The mobile application is an improvement to paper form data collection as it automates the decision-making process for surveillance officers, allows for more robust data collection, and increases efficiency in real-time reporting. Surveillance data collected is uploaded to a cloud-based server where surveillance data can be viewed and managed by the SDAH leader, DAH, and other approved users. From October 2019 through December 2019, COHWs had the option to use either the REACT electronic system or paper-based data collection. Electronic and paper-based data were merged at the end of the study period in SAS version 9.3.

### 2.5. Surveillance case definitions

A case definition was developed to assign a case status to animals investigated through VARSP (Box 1).

BOX 1 Vietnam animal rabies surveillance program case definitions*.

- **Confirmed**
  - If the test results were positive for rabies by DFA or RTPCR
- **Probable**
  - If the animal was not tested for rabies and did not pass quarantine, AND
    - If the animal exposed two or more people or animals and showed 1 or more signs of rabies, OR
    - If the animal showed three or more signs of rabies (hypersalivation, paralysis, lethargy, abnormal vocalization, or unprovoked abnormal aggression), OR
    - Regardless of exposures or symptoms, if the animal died in quarantine, OR
    - If the animal showed one or more signs of rabies and was lost or left during quarantine, OR
    - If the animal showed one or more signs of rabies but was killed or died prior to assessment, OR
    - If the animal was killed or died prior to assessment and exposed two or more people or animals
- **Suspect**
  - A case that is compatible with the WHO clinical case definition of animal rabies or any case reported by a rabies bite center (9)
- **Not a Case**
  - If the test results were negative, OR
  - If the animal was healthy after 10 days in quarantine

*CAVEAT: for subset of observations where investigations were pre-emptively ended

- **Probable**: If animal showed abnormal signs, was reported as not healthy, and exposed 2 or more humans or animals
- **Suspect**: If otherwise

### 2.6. Data collection and analysis

Investigation and diagnostic data collected between March 2016 through December 2019 were compared with historical records of animal rabies cases and dog vaccination data collected by DAH dating back to January 2012. Data were entered into a Microsoft Access database or downloaded from the REACT server as a .csv file and exported to SAS software (version 9.3, SAS Institute Inc., Cary, NC, US). Univariate, descriptive analyses for temporal and spatial trends were conducted. Odds ratios (OR) and conditional maximum likelihood estimate tests of associations between clinical signs and case definitions (i.e., confirmed case, probable case, suspect case, or non-case) were examined to validate the case definition algorithm. A probabilistic risk analysis similar to that described by Medley et al., and published in the WHO Technical Report Series for Rabies, was conducted to characterize the risk of rabies death, assuming no PEP was initiated by exposed persons, under varying rabies exposure scenarios (9, 15, 16). A formal waiver was obtained from the CDC National Center for Emerging Zoonotic Infectious Diseases human subject’s advisor; this work was deemed exempt, non-research.

## 3. Results

**Figure 2:**
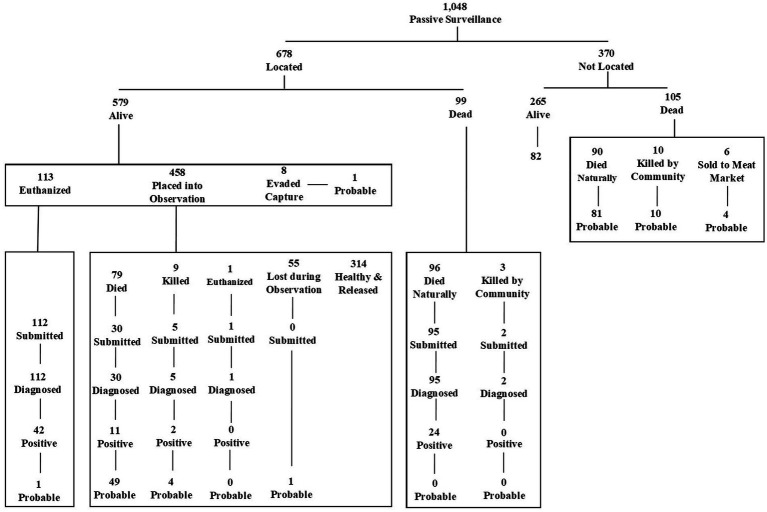
Outcomes of investigations and diagnoses of animals investigated through the Vietnam Animal Rabies Surveillance Program, Phu Tho Province, March 2016 – December 2019.

During the 46 months of VARSP data included in this analysis, 1,048 investigations were conducted which identified 79 (8%) confirmed rabies cases and 233 (22%) probable cases (Table 1). The majority of investigations conducted on high-risk (confirmed and probable cases) cases were in dogs (95.5%). Other animals investigated included 66 cats, 1 cow, and 4 unnamed animal species. Among animals investigated, 245 were submitted for testing and 79 (32%) were confirmed positive (Figure 2). Of all confirmed cases, 77 (97%) were dogs, 1 (1%) was a cat, 1 (1%) was an unspecified animal species (incomplete data on a paper form). Investigations were reported from multiple sources including human health sectors (*n* = 859), animal health sectors (*n* = 117), and the public (*n* = 43). The majority of confirmed and probable rabid animals were reported from the human health sector (87%).

**Table 1:** Rabies case status by animal species, Phu Tho Province, Vietnam March 2016 – December 2019.

Case definition	Confirmed *N* (%)	Probable *N* (%)	Suspect *N* (%)	Non-Case *N* (%)	Total
Dog	77 (8%)	221 (23%)	230 (24%)	449 (46%)	977
Cat	1 (2%)	11 (17%)	24 (36%)	30 (45%)	66
Other	1 (20%)	1 (20%)	2 (40%)	1 (20%)	5
**Total**	**79 (8%)**	**233 (22%)**	**256 (25%)**	**480 (46%)**	**1,048**

**Figure 3:**
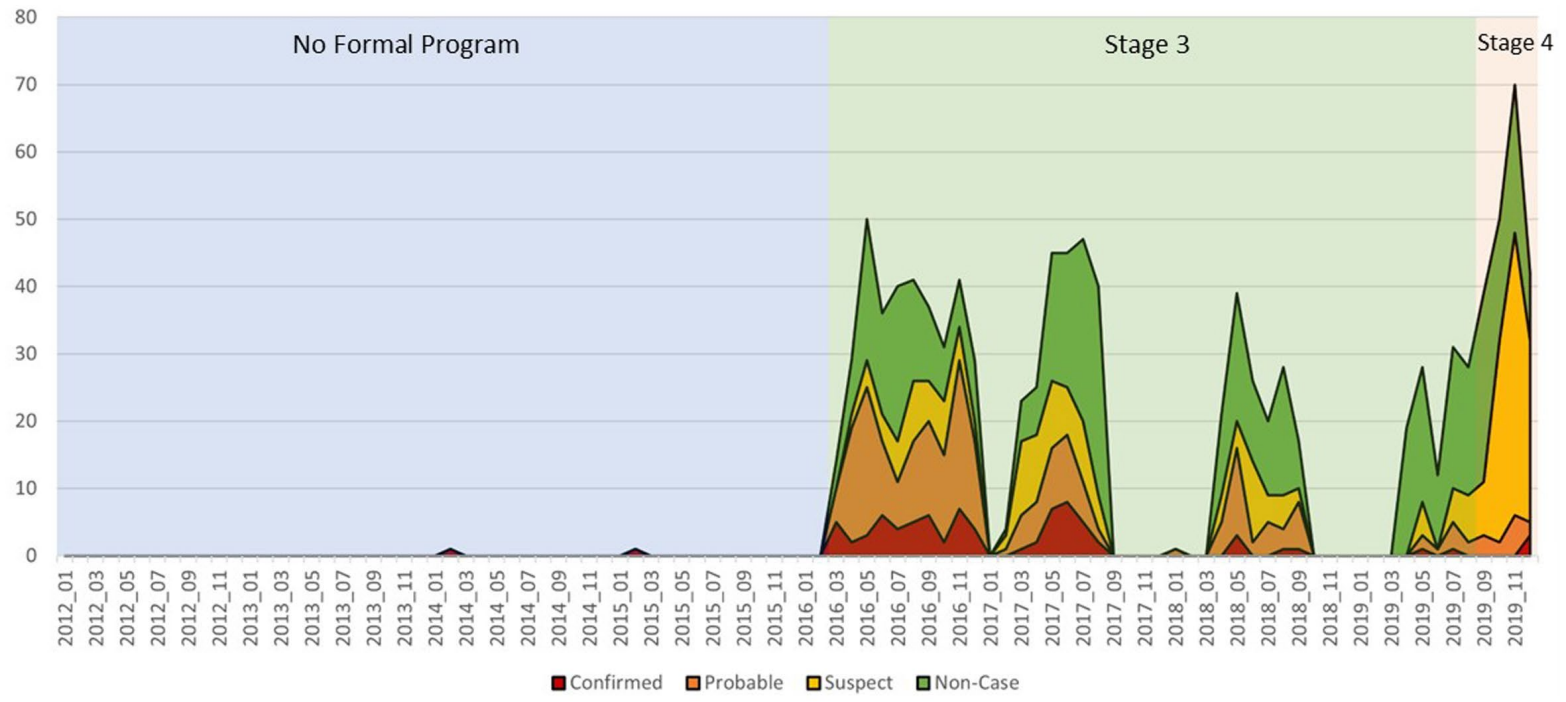
Animal rabies case statuses by date of diagnosis, Phu Tho, January 2012–December 2019.

Prior to the implementation of VARSP, between 2012 and 2015, there was an average of one rabies case investigation per year, resulting in an average of one confirmed or probable rabid animal detected (Figure 3). After the introduction of IBCM, there were, on average, 262 rabies investigations conducted per year (262-fold increase) and 20 laboratory confirmed cases detected (7%) resulting in an over 78-fold increase in annual case detection after implementation. External funding from US CDC supported 796 investigations over the 46-month study period. As a result of the training, and supported by local funding, an additional 300 investigations were conducted by local DAH officials over the same time period. This represents a 28.6% increase in rabies surveillance activities as an indirect benefit of this externally funded program.

**Figure 4:**
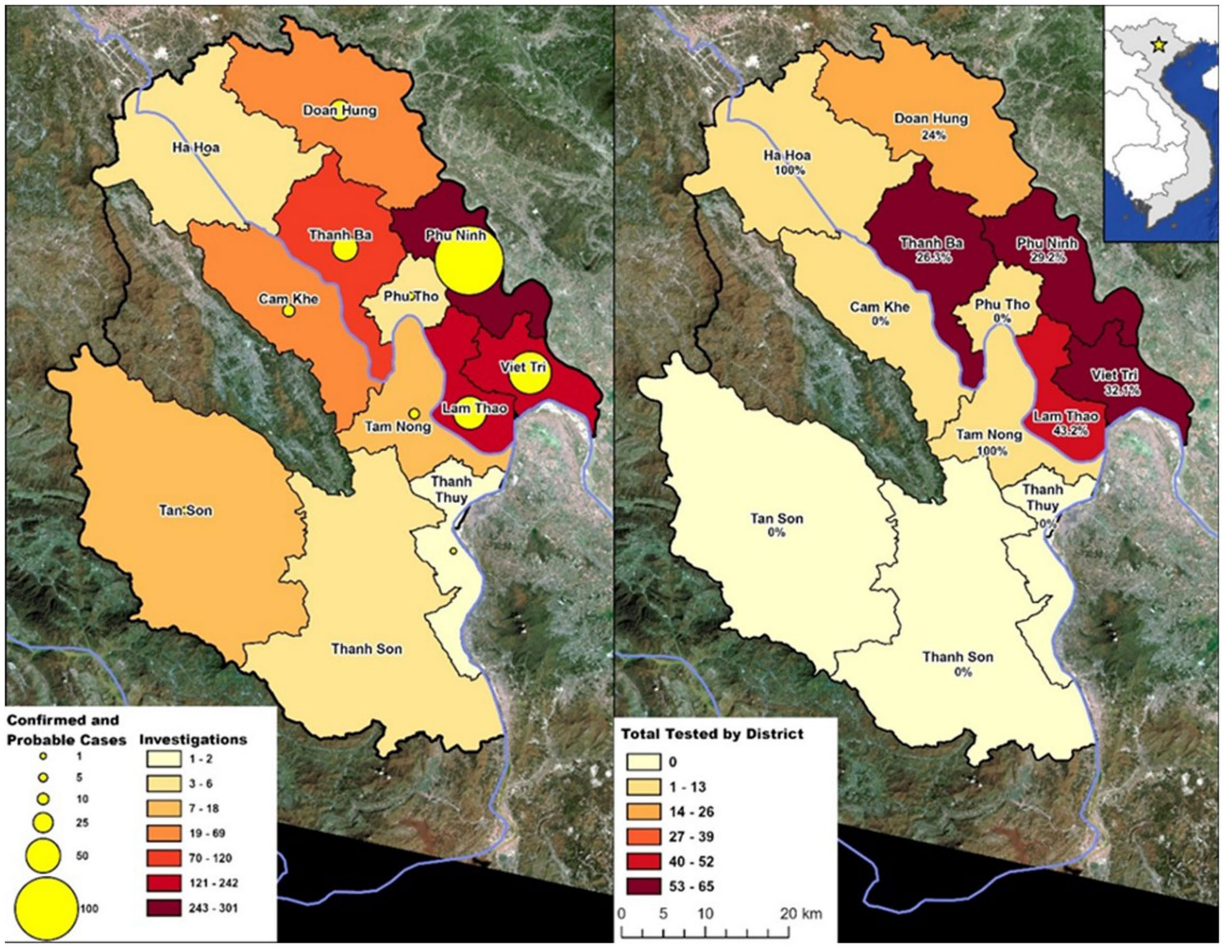
Animal rabies activities in Phu Tho Province by district, March 2016–December 2019. Percent positive of tested samples are indicated below district name. Data retrieved from usgs.gov. Landsat 8–9 OLI/TIRS C2 L2 (09-30-2019).

VARSP investigations were conducted in 12 of 13 districts in Phu Tho, with rabid animals reported from 8 districts (Figure 4). The six VARSP-focus districts accounted for 91% of investigations conducted and identified 76 rabid animals. Confirmed and probable cases made up 50% (*n* = 3) of investigations in Phu Tho city, 38% (*n* = 26) of investigations in Doan Hung district, 37% (*n* = 110) of investigations in Phu Ninh district, 30% (*n* = 64) of investigations in Viet Tri city, 29% (*n* = 35) of investigations in Thanh Ba district, and 20% (*n* = 48) of investigations in Lam Thao district.

Often-cited signs of rabies (hypersalivation and paralysis) were not commonly recognized among confirmed rabid dogs by the COHWs, at 10 and 5%, respectively (Table 2). Lethargy was commonly noted among rabid animals (32%) but was not significantly associated with animals confirmed as rabid, as it was common among all dogs investigated by COHWs (*p* = 0.22). Odds ratios are significantly higher for most clinical signs in confirmed rabid animals and associations between rabies and these clinical factors had a diminishing relationship with case status (probable, suspect, and non-case). The highest odds of an animal having rabies were observed when they had at least 2 symptoms (OR = 7.8), when they exhibited hypersalivation (OR = 6.1), or when they displayed unexplained aggression (OR = 2.9).

**Table 2:** Clinical signs observed during investigation of suspected rabid animals by case status, Phu Tho Province, March 2016 – December 2019.

	Confirmed			Probable			Suspect			Non-case		
Clinical signs	*N*	%	OR	*N*	%	OR	*N*	%	OR	*N*	%	OR
Aggression	52	66%	2.9**	123	53%	2.3**	55	21%	0.9	111	23%	Ref
Biting	35	44%	1.8**	79	34%	1.4*	47	18%	0.7	119	25%	
Hypersalivation	8	10%	6.1**	6	3%	1.6	1	0%	0.2	8	2%	
Paralyzation	4	5%	3.0	7	3%	1.8	0	0%	–	8	2%	
Lethargy	25	32%	1.4	94	41%	1.7**	5	2%	0.1**	112	23%	
**Total**	**79**	**100%**		**232**	**100%**		**257**	**100%**		**481**	**100%**	
0	13	16%	0.3**	49	21%	0.4**	192	75%	1.4**	259	54%	
1	43	54%	1.3	136	59%	1.4*	62	24%	0.6**	203	42%	
2	23	29%	7.8**	47	20%	5.4**	3	1%	0.3	18	4%	

*Indicates significance at 0.05. **Indicates significance at 0.01.

Of all confirmed cases, 52% (*n* = 41) fit the probable clinical case definition and 48% (*n* = 38) fit the suspected clinical case definition. Additional case definition validation occurred through the evaluation of dogs with known outcomes. The probability of a dog, identified by a COHW as meeting the clinical case definition for rabies (probable), being confirmed for rabies through laboratory testing, if such testing were conducted, was 49%. The probability of a dog meeting the suspected rabies case definition being laboratory-confirmed for rabies, if testing were performed, was 8%. This decline in probability further supports the case definitions used in Vietnam.

As part of the bite investigation, COHWs document each bite occurrence. Data collected show a high rate of PEP received regardless of final animal outcome or risk-level of the exposure (Table 3). After a dog bite that was reported to VARSP, 97.2% of people exposed received PEP regardless of case investigation findings. Among all human exposures to dogs, 97.4% of people received PEP. Of those who received PEP, 22.3% were found to have been at low risk for rabies and 36.3% were found to be at no risk for rabies. Of those with no risk for rabies, 96.6% received PEP.

**Table 3:** Human exposures to suspected rabid animals by case status and species, Phu Tho Province, March 2016 – December 2019.

Animal	Case definition	Bites				Scratches/other				Total persons exposed			
		People bitten		People received PEP		People scratched		People received PEP		Total exposures		Received PEP	
		*N*	%	*N*	%	*N*	%	*N*	%	*N*	%	*N*	%
Dog	Confirmed	107	11.1%	103	96.3%	1	7.7%	1	100.0%	108	11.1%	104	96.3%
	Probable	291	30.2%	290	99.7%	5	38.5%	5	100.0%	296	30.3%	295	99.7%
	Suspect	215	22.3%	207	96.3%	3	23.1%	3	100.0%	218	22.3%	210	96.3%
	Non-case	350	36.3%	338	96.6%	4	30.8%	4	100.0%	354	36.3%	342	96.6%
**Total**		**963**	**100.0%**	**938**	**97.4%**	**13**	**100.0%**	**13**	**100%**	**976**	**100.0%**	**951**	**97.4%**
Other	Confirmed	2	2.8%	2	100.0%	0	0.0%	0	0.0%	2	2.6%	2	100.0%
	Probable	14	19.7%	14	100.0%	3	60.0%	3	100.0%	17	22.4%	17	100.0%
	Suspect	25	35.2%	23	92.0%	1	20.0%	1	100.0%	26	34.2%	24	92.3%
	Negative	30	42.3%	28	93.3%	1	20.0%	1	100.0%	31	40.8%	29	93.5%
	**Total**	**71**	**100.0%**	**67**	**94.4%**	**5**	**100.0%**	**5**	**100.0%**	**76**	**100.0%**	**72**	**94.7%**
Total	Confirmed	109	10.5%	105	96.3%	1	5.6%	1	100.0%	110	10.5%	106	96.4%
	Probable	305	29.5%	304	99.7%	8	44.4%	8	100.0%	313	29.8%	312	99.7%
	Suspect	240	23.2%	230	95.8%	4	22.2%	4	100.0%	244	23.2%	234	95.9%
	Negative	380	36.8%	366	96.3%	5	27.8%	5	100.0%	385	36.6%	371	96.4%
	**Total**	**1,034**	**100.0%**	**1,005**	**97.2%**	**18**	**100.0%**	**18**	**100.0%**	**1,052**	**100.0%**	**1,023**	**97.2%**

We conducted a probabilistic risk assessment to evaluate the risk of death as a result of being exposed to rabies virus based on data collected over the course of an IBCM investigation (Table 4). Dogs that exposed multiple people (*n* = 37) posed a higher risk for rabies (73%). The exposure with the highest risk of death was a bite to the head or neck by a dog that exposed multiple people (32.8%). For dogs that were healthy and available for quarantine (*n* = 147), the risk for a bite victim to succumb to rabies was low (0.7%) and there is no risk to the bite victim when dogs were healthy after the 10-day quarantine period. The probability of developing rabies was low (<1%) when there were minor bites with no breaks in the skin. If a person was bitten on an extremity by a healthy dog that was available for quarantine, the risk that they would develop rabies in the absence of rabies PEP was 0.03 (three human rabies deaths per 10,000 people under these conditions). Assuming the typical five-dose vaccination series costs $75 USD, this equates to a cost of $220,588.24 USD per death averted, which is 26x higher than the WHO cost-effectiveness indicator of 3-times the GDP *per capita* (17). In comparison, a person who was bitten on an extremity by a dog exhibiting at least one clinical sign of rabies, the cost per death adverted is $7,500 USD, which is below the WHO cost effectiveness indicator.

**Table 4:** Probability of succumbing to rabies virus infection after exposure to dogs in Phu Tho Province, 2016–2019.

Exposure consideration	Probability of death based on level of exposure	Information collected at time of exposure							Quarantine or testing	
		Dog exposed multiple people	Dog dead within 10 days of exposure	Unowned Dog	Dog bite was not provoked (Aggressive)	Dog not vaccinated	Dog is showing >1 clinical sign of rabies	Dog healthy and available for quarantine	Dog healthy at 10 days post-bite	Tested negative
Bite(s) to head/neck	45%	32.8%	25.3%	20.5%	15.1%	10.7%	9.4%	0.3%	0.0%	0.0%
Multiple severe bite wounds	27.50%	20.1%	15.5%	12.5%	9.2%	6.6%	5.7%	0.2%	0.0%	0.0%
Bites to young children	27.50%	20.1%	15.5%	12.5%	9.2%	6.6%	5.7%	0.2%	0.0%	0.0%
Bites to extremities	5.00%	3.6%	2.8%	2.3%	1.7%	1.2%	1.0%	0.0%	0.0%	0.0%
Minor bites (no break in skin)	1.00%	0.7%	0.6%	0.5%	0.3%	0.2%	0.2%	0.0%	0.0%	0.0%
Probability the dog has rabies		73.0%	56.3%	45.5%	33.6%	23.9%	20.8%	0.7%	0.0%	0.0%
Confirmed		27	27	5	45	63	59	1	0	0
Eligible dogs (*n*)		37	48	11	134	264	283	147	285	61

Probabilities are shaded on a scale from green, yellow, orange, red to represent the range of risk with green representing the lowest risk and red representing the highest risk.

## 4. Discussion

### 4.1. Establishment of routine surveillance

The rabies surveillance system implemented in Phu Tho Province, Vietnam is based on the World Health Organization (WHO)-supported IBCM approach for investigation and data recording that has also been implemented previously in dog-mediated rabies endemic countries (18, 19). IBCM integrates data collection and reporting at the community level of animal and human health sectors to form a cohesive response to managing animal bites and PEP recommendations. Outcomes of this One Health approach can lead to reduced rabies transmissions in communities through rapid response, animal evaluation, contact tracing, and implementation of control measures (18, 19). Accomplishing a functional IBCM program in Vietnam entailed the training of health care and public health workers, establishing laboratory capacity, raising community awareness of rabies, and development of systems and protocols to ensure that investigation results involving animal bite victims were shared with PEP providers in a timely manner. As a result of this One Health approach, fostering collaboration between the human health sector and the animal health sector, COHWs were able to investigate 859 bite cases which made up the majority of the investigations conducted in the VARSP-focused districts. This highlights the importance of multi-sector communication to bolster animal rabies surveillance systems. Key outcomes of these efforts were two-fold: (1) to reduce the rabies burden and human lives lost, and (2) more effective and efficient use of human and dog vaccines.

The establishment of a case definition is a foundational component of any surveillance system as it standardizes the criteria to identify cases and defines when public health and animal health interventions are indicated. Animals that test positive fall into a standard “confirmed” case definition and animals that test negative or pass the 10-day quarantine period fall into a standard “non-case” definition. However, many animals investigated for bites in rabies endemic countries have no definitively known final health outcome, often due to low rates of animal ownership and proclivity for owned dogs to roam freely among other factors. Animals that are not available for testing or quarantine, or that test inconclusively, are often harder to classify and fall into “probable” or “suspect” case definitions. These definitions have multiple levels of considerations and have been shown to be inconsistently applied across rabies programs, particularly when depending upon paper-based or non-standardized information collection systems (8). Odds ratios among rabid animals in VARSP were significantly higher for clinical signs such as aggression, biting, and hypersalivation, but these associations were progressively reduced for lower-risk case status (probable, suspect, non-case). This decrease in associations supports the case definitions used for probable and suspect rabid animals by VARSP.

The surveillance system implemented in Vietnam is based on the same model that was adopted in Haiti, with slightly different case definitions reflecting input from the National Rabies Program. As a result, a higher proportion of investigations in Vietnam resulted in a probable case definition when compared to the results in Haiti (22% vs. 4.6%). These differences can be attributed to the different case definitions used in each country. The probable case definition used in Vietnam covers a broad scope of scenarios to account for the lack of availability of the animal for testing, clinical signs of rabies during the assessment, quarantine result, and the number of human and animal exposures for probable cases. Haiti’s probable case definition differs in that the number of human and animal exposures are not included in the algorithm to define a probable case (9, 18). Among animals that could be tested, Vietnam’s percent positivity among dogs meeting the probable case definition was consistent with the results observed in Haiti (49% vs. 50%) which may suggest that among animals eligible for testing and under the structure of this IBCM approach, there is a consistent likelihood of detecting rabies virus infections. The variation in case definitions between the two countries which followed the same IBCM model highlights the importance of designing a case definition that supports the specific program, but also showcases how important case definitions are when analyzing and interpreting surveillance data.

While some variables are highly associated with rabies case status in biting dogs, no single variable alone was adequate for the basis of a risk assessment and determining if PEP is indicated (15, 20). Rabies cases were documented among dogs with a reported history of vaccination, among owned dogs, and among dogs originally considered low risk at the time of assessment. As seen in other studies, a combination of risk factors must be considered to make an accurate assessment of the rabies status of a biting dog (15, 19). In this analysis, biting dogs that are assessed by an animal health professional to be healthy and are available for in-home quarantine by their owners were highly unlikely to develop rabies and represent a very low risk of rabies transmission to their bite victims (0.03%). These findings are similar to those in Haiti where bites from dogs that were assessed as healthy and available for quarantine represented a 0.05% risk of rabies to the bite victim and further supports the WHO IBCM protocol (15). WHO recommends that PEP be provided based upon an appropriate risk assessment by a health professional, where available (9). The results of this evaluation show that in the Phu Tho Province, the animal health professional’s assessment of the animals was accurate and when combined with the ability to self-quarantine a healthy dog, the risk of delaying PEP for the majority of bite victims is negligible. This analysis also highlights that treating all persons with PEP for these low-risk exposures is not cost-effective. At least two countries have shown that a protocol in which persons delayed PEP while healthy dogs were under quarantine would present almost no risk to the bite victim, as they would receive PEP in the rare situation if the dog developed rabies during the 10-day quarantine period (15, 19).

### 4.2. Rabies burden

During the study period, VARSP was able to identify 79 laboratory confirmed rabid animals. This is a dramatic increase compared to 2012–2015 where only two confirmed cases were reported. There are three time periods with no or very few investigations (Figure 3). This should not be interpreted as a decline in suspect rabid dogs during these time periods, but rather an indicator of the funding mechanisms not being available and surveillance efforts temporarily halted. Also during this time was a decline in the proportion of high-risk cases (6-fold decrease in proportion of high-risk cases from 2016 to 2019/51.6 vs. 8.5%). During 2016–2017, 40 high-risk dogs were euthanized and removed from the community, and this may have limited the spread of rabies in the community. However, this impact is likely to be small and does not fully account for the decline in high-risk cases which were observed. Dog vaccination efforts declined in 2016, after cessation of a WOAH and FAO-supported dog vaccination program which provided free vaccines to dog owners (5). During the study period, routine vaccination campaigns were held by DAH, but dog owners were charged approximately $1 per rabies vaccine and vaccination coverages by this approach were thought to reach only 40% of the dog population. The cause of the apparent reduction in high-risk rabies cases in Phu Tho during the study period is unknown but could reflect enzootic multi-year cycles of disease transmission (21–23).

### 4.3. Benefits of routine animal rabies surveillance

The IBCM system implemented in Vietnam is a WHO-supported approach to rabies surveillance that integrates data collection and reporting at the community level with animal and human health sectors to form a cohesive response to animal bites. It accomplishes this by limiting rabies transmission in communities through rapid response, animal evaluation, contact tracing, and implementation of control measures; this entails the training of health care and public health workers, raising community awareness of rabies, and referring animal bite victims to existing centers where post-exposure prophylaxis (PEP) can be received.

In March 2018, an official decision on the implementation of Event Based Surveillance (EBS) in Vietnam was issued (24). Accordingly, dog bites became a signal required to be reported to the health system for early warning and response. In addition, animals (especially dogs) with signs consistent with rabies are required to be notified to local human health or animal health authorities. If implemented properly, EBS will enhance the coordination and more frequent information exchange between the animal and human health sectors, improving the prospects for expanded implementation of IBCM, coordinated rabies surveillance and outbreak response efforts, following a One Health approach.

Vietnam is one of only a few rabies endemic countries in Southeast Asia where PEP is widely available. The majority of medical centers follow the Essen 5 dose schedule while others deliver vaccine using the Updated Thai Red Cross 8-dose schedule (25). Using the surveillance data collected over the course of the VARSP operation, PEP could have been averted for 366 people (92 people per year) and safely delayed for 150 people who were exposed to healthy dogs available to be quarantine. However, PEP was still regularly administered under this pilot program as WHO does not recommend risk-based PEP unless there is a functional surveillance system in place to ensure appropriate risk assessment and animal follow up is conducted. PEP in Vietnam costs $75 per person for the complete series. This represents a potential PEP savings among the study population of $6,900. Future IBCM program implementation should consider using investigation results to inform PEP decisions in low-risk situations where PEP can safely be delayed. The cost-savings in PEP could be used to supplement IBCM operational costs or complementary dog vaccination programs (26).

The transition of Vietnam’s Animal Rabies Surveillance Program from a paper-based system to an app-based system (REACT) offers several advantages. Due to the complex nature of rabies investigations, using an app-based approach removes some of the time-sensitive decisions that COHWs must make pertaining to the risk of the animal having rabies and the appropriate PEP recommendation for exposed individuals and potential euthanasia decisions for animals. Interviews with surveillance officers in Haiti who used both paper-based and app-based systems determined that the majority of surveillance officers agreed with statements pertaining to the ease of use, timeliness of report submission, rabies risk assessment, and timeliness of data analysis (14). In addition, this system allows for more detailed data collection that would not otherwise be documented in a paper-based system. Lastly, the use of an app-based system allows for more timely and impactful data sharing with local partners, national programs, and international collaborators. Having access to near real-time surveillance allows for more efficient management of rabies surveillance in Vietnam and provides a strong impetus to continue funding rabies prevention activities. Since 2020, DAH has trained an additional 369 COHWs on the VARSP and REACT module for expanded rabies surveillance.

### 4.4. Limitations

As seen in similar IBCM implementation scenarios, the passive surveillance system in the Phu Tho Province relies on notifications from medical centers and the community to initiate data collection. The human population of the study area in 2016 was 621,356. Estimates by Hampson et al. estimate the annual bite rate in Vietnam as 378/100,000 (1). By these estimates, we should expect to see approximately 2,349 bites meaning approximately 88% of bites are not being reported or investigated. This is likely due to multiple factors, including the lack of healthcare seeking, periodicity in VARSP operations, and missed opportunities for case notifications. The impact of under-detection of bite cases on our knowledge of rabies epidemiology in Pho Tho is unknown. In addition, the system is designed to investigate dogs, the primary rabies reservoir in Vietnam. As a result, very few spillover species were investigated under this protocol, resulting in poor characterization of non-dog rabies transmission in the program area.

## 5. Conclusion

The pilot IBCM model used in Phu Tho Province showed promising results for being a cost-effective strategy to improve rabies surveillance, with a   > 78-fold increase in annual case detection after implementation. The risk for a person bitten by an apparently healthy dog to develop rabies in the absence of rabies PEP was very low, which supports WHO guidance to delay PEP for this category of bite victims, when trained animal assessors are available to evaluate the offending dog. While WHO recommends a risk-based approach under the aforementioned conditions, the ultimate decision to pursue PEP should be made between the practitioner and the patient. The recent adoption of an electronic IBCM system is likely to expedite adoption of this IBCM program to other Provinces to further improve rabies surveillance and resulting health outcomes nation-wide.

## Data availability statement

The raw data supporting the conclusions of this article will be made available by the authors, without undue reservation.

## Ethics statement

The animal study was approved by Institutional Animal Care and Use Committee, US Centers for Disease Control and Prevention. The study was conducted in accordance with the local legislation and institutional requirements.

## Author contributions

CV, MP, DN, TXN, TTN, LO, TDN, ON, TD, AD, and LN contributed to conception and design of the study. RW and RDT (REACT Development Team) organized the database. YR, CV, SB, and RW performed the statistical analysis. YR wrote the first draft of the manuscript. YR, CV, LO, RW, and RDT (REACT Development Team) wrote sections of the manuscript. All authors contributed to manuscript revision, read, and approved the submitted version.

## REACT Development Team

Andrew Luke Gamble, Mission Rabies; Fred Lohr, Mission Rabies; Luke Gamble, Mission Rabies.
