# Supplementary material for: Measuring the impact of an integrated bite case management program on the detection of canine rabies cases in Vietnam
Source: Front Public Health. 2023 Oct 18;11:1150228. doi: 10.3389/fpubh.2023.1150228 (PMC10619753; doi:10.3389/fpubh.2023.1150228)
Supplement: Supplementary file 1 [file Table_1.docx]

## APPENDIX

## 13. Standard Operating Procedures: Euthanasia for Suspect Rabid Animals

*Adapted from Humane Society International

Justification:

General anesthesia may be impractical, and as such, we will suggest other methods of euthanasia listed under AVMA guidelines:

“Although unacceptable and condemned when used in unanaesthetized animals, the use of a supersaturated solution of potassium chloride injected intravenously or intracardially in an animal under general anesthesia is an acceptable method to produce cardiac arrest and death.”

Method:

This method may be performed *intracardially in conjunction with general anesthesia only.*

Although intravenous injection is reported in protocol documents, it is not favored for euthanasia. Reasons for this include:

1. It requires a venous access, which is more difficult and more stressful for the animal
2. It will take more time to reach the heart, especially if the animal is under heavy sedation (reduced blood pressure)
3. It could be painful

Guidelines:

Classification: Agents causing hypoxia

Storage: Room temperature

Mode of action: Cardiotoxic. Direct depression of cerebral cortex, subcortical structures, and vital centers secondary to cardiac arrest.

Rapidity: Rapid

Ease of performance: Relatively easy when performed by an experienced person

Dose: Rapid intracardiac administration of 1 to 2 mmol/kg of body weight will cause cardiac arrest

Commercially available products: Potassium Chloride Injectable (Abbott), 40 mEq/20ml (2mEq/ml)

Safety for personnel: Avoid accidental human exposure

Human/Animal risk factor: Preferred injectable technique to reduce the risk of toxicosis for predators or scavengers in situations where carcasses of euthanatized animals may be consumed.

Species suitability: Most species.

Efficacy and Comments: Highly effective

Advantages:

- Not a controlled substance
- Easy to purchase and inexpensive
- The carcass is potentially less toxic for scavengers and predators in cases where carcass disposal is impossible or impractical

Disadvantage:

- Rippling of muscle tissue and clonic spasms may occur on or shortly after injection

Recommendations

- It is of utmost importance that personnel performing this technique are trained and knowledgeable in anesthetic techniques, and are competent in assessing anesthetic depth appropriate for administration of potassium chloride
- Administration of potassium chloride requires animals to be in a surgical plane of anesthesia characterized by loss of consciousness, loss of reflex muscle response, and loss of response to noxious stimuli.

*Figure 17: Euthanasia Protocol Summary*

| **SEDATION**   - Xylazine 2ml (1 bottle of 100mg/ml) + Ketamine 10ml (1 bottle of 100mg/ml) - **1 – 2 mL / 10 kg intramuscular injection**    - given to effect, to produce complete anesthesia   **LETHAL INJECTION**   - Potassium Chloride Injectable   - 40 Eq/20ml (2mEq/ml) - Dose: 1 to 2 mmol/kg   - 1 mmol/L = 74.5 mg - **1 ml/kg intracardiac injection**   - Typically, 10 – 20 mL needed |
| --- |

### 13.1 Other Acceptable Methods of Euthanasia:

Other acceptable methods for euthanasia area possible for consideration. If facilities and supplies are available, the following tables describe acceptable and unacceptable methods of euthanasia.

| *Figure 18: Other acceptable methods of euthanasia*   \| **Species** \| **Acceptable*** \| **Conditionally acceptable∞** \| \| --- \| --- \| --- \| \| Cats \| Barbiturates, inhalant anesthetics, potassium chloride in conjunction with general anesthesia, Carbon dioxide, Carbon monoxide \| Nitrogen, argon \| \| Dogs \| Barbiturates, inhalant anesthetics, potassium chloride in conjunction with general anesthesia, Carbon dioxide, Carbon monoxide \| Nitrogen, argon \| \| Ruminants \| Barbiturates, potassium chloride in conjunction with general anesthesia, Penetrating captive bolt \| Chloral hydrate (IV after sedation), gun shot \| \| Swine \| Barbiturates, potassium chloride in conjunction with general anesthesia, Carbon dioxide, Penetrating captive bolt \| Inhalant anesthetics, Carbon monoxide, chloral hydrate (IV after sedation), gun shot \| \| Free-ranging wildlife \| Barbiturates IV or IP, inhalant anesthetics, potassium chloride in conjunction with general anesthesia \| Carbon dioxide, Carbon monoxide, nitrogen, argon, gun shot \|   ** Acceptable methods are those that consistently produce a humane death when used as the sole means of euthanasia.*  *∞Conditionally acceptable methods are those that by the nature of the technique or because of the greater potential for operator error or safety hazards might not consistently produce humane death.*  *Modified from: “AVMA Guidelines for the Euthanasia of Animals”* |
| --- | --- | --- | --- | --- | --- | --- | --- | --- | --- | --- | --- | --- | --- | --- | --- | --- | --- | --- |

### 13.2 Conditionally acceptable agents and methods of euthanasia

Conditionally acceptable agents and methods of euthanasia—characteristics and modes of action

| *Figure 19: Conditionally acceptable agents and methods of euthanasia*   \| **Agent** \| **Mode of Action** \| **Rapidity** \| **Ease of performance** \| **Safety for personnel** \| **Species** \| **Efficacy/Comments** \| \| --- \| --- \| --- \| --- \| --- \| --- \| --- \| \| Potassium Chloride  *****Must be used in conjunction with general anesthesia only (intracardially or IV^1^)** \| Hypoxia; Depression of the cerebellar cortex secondary to cardiac arrest \| Rapid \| Requires training for: remote injection anesthesia; ability to give Intracardially or IV \| Anesthetics may be hazardous if humans exposed \| Most species \| Highly effective; muscles spasms may be observed \| \| Barbiturates \| Hypoxia; Depression of the cerebellar cortex \| Rapid onset of anesthesia \| Animal needs restraint, ability to give IV \| Human abuse potential \| Most species \| Highly effective \| \| Inhalant anesthetics \| Hypoxia; Depression of the cerebellar cortex \| Moderately rapid, animal may become excited during induction \| Closed container or mask \| Proper ventilation required \| Dogs, cats, wildlife \| Highly effective if correctly exposed \| \| Carbon dioxide (bottled gas only) \| Hypoxia; Depression of the cerebellar cortex \| Moderately rapid \| Closed container \| Minimized hazard \| Small dogs, cats, swine, some wildlife \| Effective; longer time required in immature/neonate animals \| \| Carbon monoxide  (bottled gas only) \| Hypoxia; prevents oxygen binding on hemoglobin \| Moderate onset, animal is unware of onset \| Requires appropriately maintained equipment \| **Extremely hazardous, toxic, difficult to detect** \| Dogs, cats, some wildlife \| Effective; equipment must be properly designed and operated \| \| Nitrogen, argon \| Hypoxia; reduces oxygen availability in blood \| Rapid \| Used in closed, rapidly filling chamber \| Proper ventilation required \| Small dogs, cats, some wildlife \| Effective but not in neonates; **other methods preferable** \| \| Penetrating captive bolt \| Physical damage to brain \| Rapid \| Requires skill, adequate restraint, proper bolt placement \| **Rabies exposure risk from potential contact with cerebrospinal fluid** \| Swine, ruminants \| Instant loss of consciousness but motor activity continues \|   ^1^IV: Intravenous  Chart modified from AVMA euthanasia guidelines |
| --- | --- | --- | --- | --- | --- | --- | --- | --- | --- | --- | --- | --- | --- | --- | --- | --- | --- | --- | --- | --- | --- | --- | --- | --- | --- | --- | --- | --- | --- | --- | --- | --- | --- | --- | --- | --- | --- | --- | --- | --- | --- | --- | --- | --- | --- | --- | --- | --- | --- | --- | --- | --- | --- | --- | --- | --- |

## Euthanasia References

Canadian Association for Laboratory Animal Medicine / L’association canadienne de la médecine des animaux de laboratoire <http://calam-acmal.org/>

Le Conseil canadien de protection des animaux en science / Canadian Council on Animal Care

<http://www.ccac.ca/fr_>

Conseil national pour les soins aux animaux d’élevage / National Farm Animal Care Council <http://www.nfacc.ca/francais>

AVMA Guidelines on Euthanasia <https://www.avma.org/KB/Policies/Documents/euthanasia.pdf>

*Lignes directrices du CCPA sur l'euthanasie des animaux utilisés en science*

<http://www.ccac.ca/Documents/Normes/Lignes_directrices/Euthanasie.pdf>

*Education and Training in Laboratory Animal Science & Welfare* <http://www.lal.org.uk/>

Jeanette O’Quin, DVM MPH, *Humane Euthanasia in Animal Shelters*, Conférence présentée à l’AMVQ le 11 mars 2012

The National Federation of Humane Societies, *Euthanasia Best Practice for Companion Animals*

<http://www.humanefederation.org/EuthanasiaBestPractice.cfm>

Rebecca H. Rhoades, D.V.M, *The HSUS Euthanasia Training Manual,* Item # 43500250

<http://marketplace.animalsheltering.org/product/the_hsus_euthanasia_training_manual>

Plumb’s, Veterinary Drug Handbook, 6^th^ Ed., Blackwell, 2008.
