# Supplementary material for: Measuring the impact of an integrated bite case management program on the detection of canine rabies cases in Vietnam
Source: Front Public Health. 2023 Oct 18;11:1150228. doi: 10.3389/fpubh.2023.1150228 (PMC10619753; doi:10.3389/fpubh.2023.1150228)
Supplement: Supplementary file 2 [file Data_Sheet_1.docx]

**Animal Rabies Investigation Form**

**Date of Notification: _ _/ _ _ / _ _ _ _ Name _______________________ Animal ID:**  |__|__| |__|__| |__||__|

D D M M N N

Medical Contact Name: ___________________________

Phone Number: _________________

Patient Name: ___________________________________

Age ___________

Phone Number: _________________

1. Reported From:

Health Department: ________________________

Hospital: _________________________________

Vet Agent: _______________________________

Veterinarian

**Notification**

Public

2. Reason for report:  Human Exposure (bite or scratch)  Sick animal  Hit by car  Other __________________

3. Type of animal:  Dog  Cat  Goat  Pig  Bat  Other ____________________________

4. Was this animal:  Owned  Stray  Food  Unknown

5. Location of animal exposure: Province_________________ District _______________ Commune__________________ Village_______________

**NOTES**:

6. Date of Investigation (dd/mm/year): __ __/__ __ /__ __ __ _ 7. Type of Investigation? ☐ Owner Phone Consultation ☐ In-Person Investigation

8. How many people were bitten by the animal? ______________ How many people were scratched by the animal? ______________

9. How many people received rabies vaccine? ___________ How many people did you refer for medical treatment? __________

10. What other animals were bitten by this animal? How many? ☐ Dog _____ ☐ Cat _____ ☐ Goat _____ ☐ Other _____

11. Was the animal located? **Yes**  Alive  Escaped capture  Dead, killed by owner  Dead, killed by public

Dead, killed by car  Dead, natural causes  Dead, unknown causes

**Investigation**

**No**   Not found  Dead, killed by owner  Dead, killed by public

Dead, killed by car  Dead, natural causes  Dead, unknown causes

**GPS:** N…………………… W...……………………..

12. Where was animal located? Province/District__________________

13. What is the animal’s age?  Puppy (<6 months)  Adult (6 months- 5 years)  Senior (> 5 years)

14. What is the animal’s sex?  Male  Female

15. Has the animal been vaccinated for rabies?  Yes, year: _________  Not vaccinated  Unknown

**NOTES**:

16. Signs of Disease:  Aggression  Biting  Hypersalivation  Paralyzed  Lethargy  Unknown

Other (specify) __________________________________________________________________________________

17. Rabies Assessment:  Healthy  Sick, signs of rabies  Sick, not rabies  Dead

Country: Vietnam Revised: 07/2/2016

Other (specify)__________________________________________________________________________________

18. Assessment Decision:  Quarantine  Euthanize  Dead  Other ____________________________________________

**Assessment**

Euthanized  Natural Causes  Other ________

Killed by Owner  Killed by Community

Died, date _________

19. Quarantine results:  Healthy after 10 days

Animal was lost  Decomposed / Burned

Body discarded  Other _______________

No

20. Was the animal submitted for testing?  Yes, date: _________

**NOTES**:

21. Date specimen received at lab: ______________ 22. Lab ID Number: ______________ 23. Date tested: ______________

**Lab**

24. Test Results:  Positive  Negative  Inconclusive

**Hospital notified, date_______________  Health department notified, date _______________**
